# Ablation of persistent atrial fibrillation based on atrial electrogram duration map: methodology and clinical outcomes from the AEDUM pilot study

**DOI:** 10.1007/s10840-023-01721-7

**Published:** 2024-01-11

**Authors:** Pietro Rossi, Filippo Maria Cauti, Marco Polselli, Michele Magnocavallo, Marta Niscola, Veronica Fanti, Luca Rosario Limite, Antonietta Evangelista, Alessandro Bellisario, Ruggero De Paolis, Simone Facchetti, Raffaele Quaglione, Gianfranco Piccirillo, Stefano Bianchi

**Affiliations:** 1 Arrhythmology Unit, Ospedale Fatebenefratelli Isola Tiberina-Gemelli Isola, Via Ponte Quattro Capi, 39, 00186 Rome, Italy; 2 Abbott Medical, Via Paracelso 20, 20864 Agrate Brianza, Italy; 3 Unité d’Arythmologie, Hôpital Privé Les Franciscaines, Nîmes, France; 4 European Hospital, Via Portuense, 700 Rome, Italy; 5 Department of Internal, Anesthesiology and Cardiovascular Sciences, Policlinico Umberto I, Sapienza University of Rome, 00185 Rome, Italy

**Keywords:** Atrial fibrillation, Catheter ablation, AEDUM, Persistent atrial fibrillation, Pulmonary vein isolation

## Abstract

**Background:**

Catheter ablation of persistent atrial fibrillation (*Ps*AF) represents a challenge for the electrophysiologist and there are still divergences regarding the best ablative approach to adopt. Create a new map of the duration of atrial bipolar electrograms (Atrial Electrogram DUration Map, AEDUM) to recognize a functional substrate during sinus rhythm and guide a patient-tailored ablative strategy for *Ps*AF.

**Methods:**

Forty *Ps*AF subjects were assigned in a 1:1 ratio to either for PVI alone (Group B_1_) or PVI+AEDUM areas ablation (Group B_2_). A cohort of 15 patients without AF history undergoing left-sided accessory pathway ablation was used as a control group (Group A). In all patients, voltage and AEDUM maps were created during sinus rhythm. The minimum follow-up was 12 months, with rhythm monitoring via 48-h ECG Holter or by implantable cardiac device.

**Results:**

Electrogram (EGM) duration was higher in Group B than in Group A (49±16.2ms vs 34.2±3.8ms; *p*-value<0.001). In Group B the mean cumulative AEDUM area was 21.8±8.2cm^2^; no difference between the two subgroups was observed (22.3±9.1cm^2^ vs 21.2±7.2cm^2^; *p*-value=0.45). The overall bipolar voltage recorded inside the AEDUM areas was lower than in the remaining atrial areas [median: 1.30mV (IQR: 0.71–2.38mV) vs 1.54mV (IQR: 0.79–2.97mV); *p*-value: <0.001)]. Low voltage areas (<0.5mV) were recorded in three (7.5%) patients in Group B. During the follow-up [median 511 days (376–845days)] patients who underwent PVI-only experienced more AF recurrence than those receiving a tailored approach (65% vs 35%; *p*-value= 0.04).

**Conclusions:**

All PsAF patients exhibited AEDUM areas. An ablation approach targeting these areas resulted in a more effective strategy compared with PVI only.

**Graphical Abstract:**

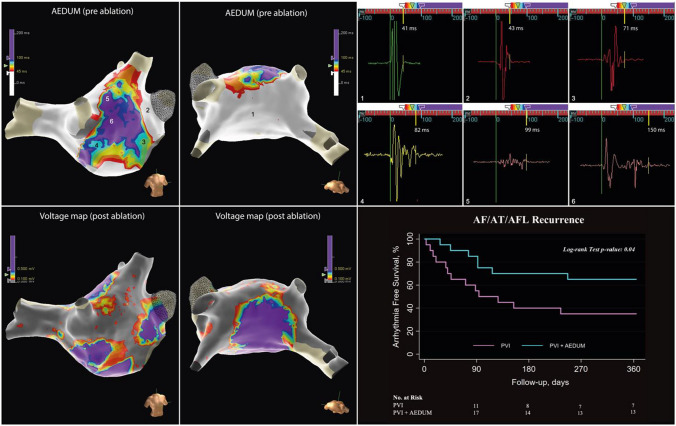

**Supplementary Information:**

The online version contains supplementary material available at 10.1007/s10840-023-01721-7.

## Introduction

Pulmonary vein isolation (PVI) is the cornerstone of catheter ablation of atrial fibrillation (AF). However, the results of PVI alone for the treatment of persistent AF (*Ps*AF) are unsatisfactory and the optimal ablation strategy is still poorly defined [1]. AF recurrences after PVI depend on the extent, localization, and degree of atrial tissue remodeling beyond pulmonary veins [2].

Several interventional strategies have been tested to identify and ablate left atrial (LA) dysfunctional areas. Anatomical approaches are usually adopted to target substrates beyond PVs, such as posterior wall (PW) isolation. A recent study reports encouraging results with this approach [3] but a randomized controlled trial do not support the empirical inclusion of (PW) isolation for ablation of persistent AF [4]. Also LA appendage, coronary sinus [5,6], vein of Marshall [7], and fibrosis areas [8–10] have been proposed as targets, but all of them had variable clinical outcomes.

In addition, ablative strategies evaluating functional substrate have been developed such as ablation of abnormal EGMs during AF [11] or identification and mapping of a functional rotational activity maintaining AF [12] or searching low voltage areas [13,14]. Although these methods are interesting because they offer tailored ablative schemes, the clinical results are still debated. In the majority of the above-mentioned studies, electroanatomical map and catheter ablation were performed during AF. The presence of extensive areas (beyond PVs) of slow conduction in pivot points has also been documented during sinus rhythm in patients with *Ps*AF [15]. Further, it has been demonstrated in animal studies that electrograms (EGMs) recorded in regions with altered anatomical/functional properties are longer in duration than in healthy regions [16]. Therefore, the analysis of EGMs characteristics during sinus/paced rhythm could better help to detect areas with pathological atrial tissue remodeling.

An electro-anatomical LA map that outlines areas with prolonged bipolar EGMs could be a descriptor of pathologic activation properties of the underlying atrial tissue, likely critical for arrhythmias’ maintenance.

The aims of the present study are as follows:

- Produce a novel left atrium electro-anatomical map based on the local duration of bipolar EGM (Atrial Electrogram Duration Map, AEDUM) registered during sinus rhythm (SR) to identify areas of slow and inhomogeneous activation in patients with *Ps*AF. A cohort of patients without a history of AF undergoing left-sided accessory pathway (LAP) ablation was used as the control group.
- To evaluate the clinical outcome of a tailored anatomical ablation scheme targeting the areas with prolonged atrial bipolar electrograms (AEDUM area).

## Methods

### Study subjects

The study population included 15 consecutive patients undergoing LAP catheter ablation that served as a control group (Group A) and 40 consecutive highly symptomatic patients undergoing *Ps*AF catheter ablation (Group B). Group B patients were alternately enrolled for PVI alone (Group B_1_) and PVI + ablation of AEDUM areas (Group B_2_). Patients who met the standard indications for *Ps*AF ablation were prospectively enrolled in this study starting in May 2019, in the Arrhythmology Unit of the Fatebenefratelli Hospital, Rome. Exclusion criteria for Group A were: age <30 years old, history of AF, thyroid disorders, and vascular/myocardial disease. Exclusion criteria for Group B were: uncontrolled hypertension, severe valvular dysfunctions, uncontrolled thyroid disease, severe chronic obstructive pulmonary disease, alcohol/drug abuse, and systemic inflammatory diseases. For all patients, antiarrhythmic medications were discontinued at least 5 half-lives before hospital admission. The study protocol was approved by the local ethics committee and written informed consent from all patients was obtained before each procedure.

### Mapping procedure

The procedures were conducted under conscious sedation in Group A, and during general anesthesia with a laryngeal mask in Group B. Patients in Group A underwent an electrophysiology study to verify the location of LAP. Transseptal puncture was performed in all patients. 3D electro-anatomic mapping around the mitral valve was performed for the AP ablation procedure, and then a complete high-density map of the left atrium was obtained.

Electrical cardioversion (ECV) was performed, and an SR map was then obtained in subjects of Group B. A second ECV was performed after PVI in the case SR was not restored or AF was triggered again during mapping. A high-density electro-anatomic map of the LA was performed only during SR either for Group A or Group B. All the LA maps were built using more than 2000 points for each map, spread across the whole atrium.

All procedures were performed using the EnSite Precision ^TM^ Mapping System and the multipolar diagnostic catheter Advisor™ HD Grid Mapping Catheter, Sensor Enabled™ (Abbott Medical).

The voltage map was created by using the EnSite Precision AutoMap Mapping Tool^TM^.

Low voltage areas (LVA) were defined as having a bipolar voltage < 0.5 mV.

### Atrial electrogram duration map

EGM duration was measured through the Ensite Precision Automatic Turbomap Tool which allowed to review SR maps and calculate the duration of each point’s EGM as the temporal distance (ms) between the first and last deflection of each bipolar endocardial EGM, applying the methodology reported in previous papers [17].

**Fig. 1:**
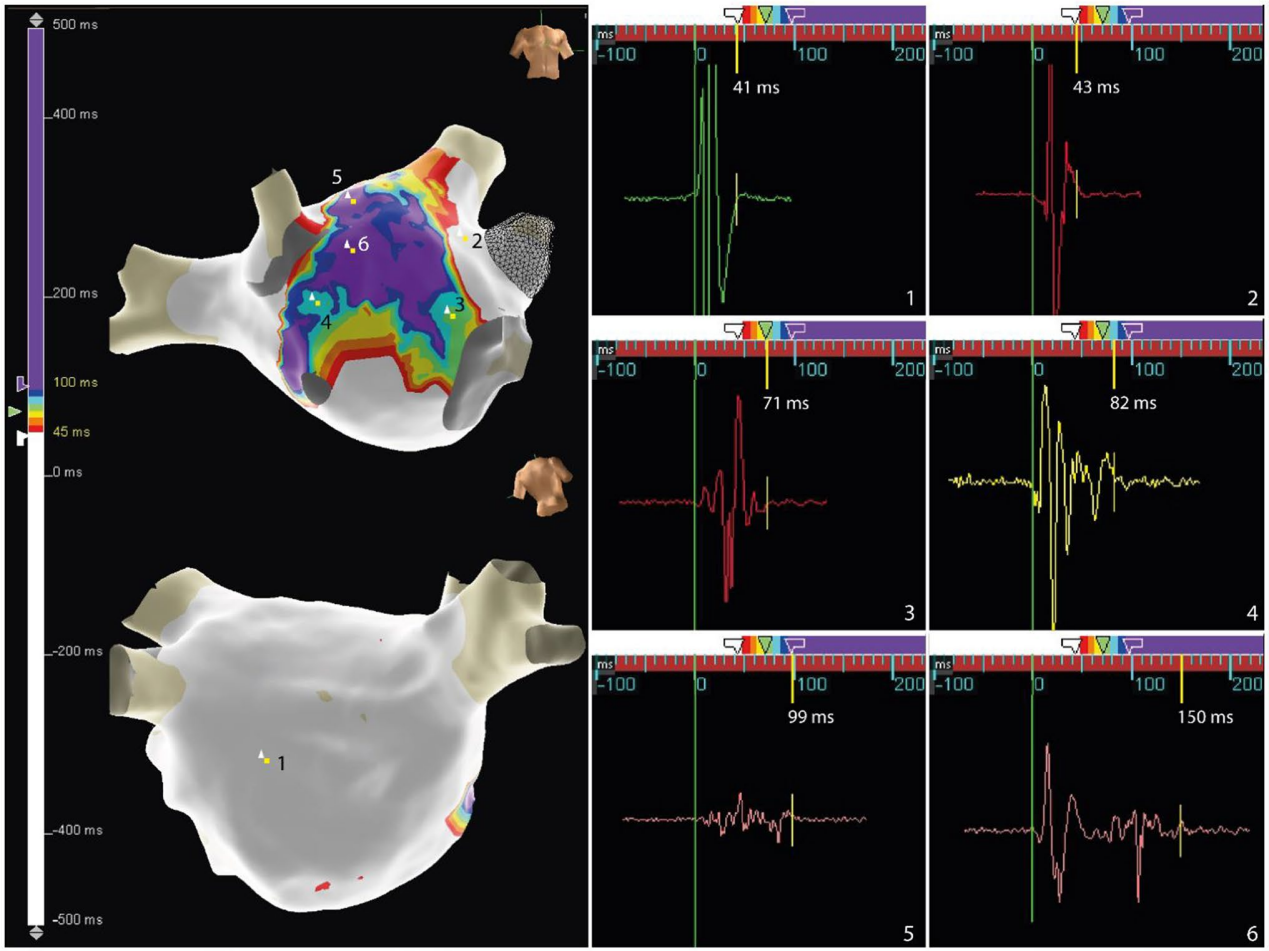
AEDUM map. The left panel shows an example of the AEDUM map in a patient with PsAF, in a slightly RAO (up) and caudal left posterior oblique (LPO, down) views. White areas exhibit potentials with a duration of <45ms, and color-coded areas exhibit potentials with a duration of ≥45ms in the septal-anterior wall. The right panels show sample EGMs obtained in this patient, in different locations on the map. Each EGM has its duration reported, and a yellow line at the end of the EGM points to the relative color on the map. Each panel is numbered, and each EGM sampling location is shown by the numbers on the map in the left panel. *AEDUM: Atrial Electrogram Duration Map; EGM: Electrogram; PsAF: Persistent Atrial Fibrillation*

Two experienced biomedical engineers (M.N. and V.F.) verified the measurement of the EGM duration intraoperatively after having completed the LA map. The points used to measure the EGM duration were carefully reviewed also offline to confirm the correct measurement. In both groups (A and B) AEDUM maps were created (Fig. 1). This map shows through a color-coded representation the distribution of EGM durations. Additional details on the mapping methodology have been reported in the supplementary materials.

We performed an interim analysis of left atrial EGM durations of patients with LAP (Group A). As reported in the Results Section, the mean EGM duration of Group A was: 34.2 ± 3.7 msec. The Empirical Rule states that 99.7% of data observed following a normal distribution lies within 3 standard deviations of the mean. Therefore, under the value of 45 msec (mean + 3 standard deviation) was included the 99.7% of normal EGM duration. Thus, a cut-off value of 45 msec was chosen to identify areas of atrial conductive dysfunction [18,19] and zones with EGMs longer than 45 msec were defined as AEDUM Areas.

### Left atrium AEDUM analysis

The LA was divided into 14 segments as previously reported [19]. The following parameters were noted for each patient: 1) the extent of the total area with voltage <0.5 mV during SR and the corresponding involved segments; 2) AEDUM areas extension and corresponding involved segments.

### Ablation procedure

In Group A, focal ablation was performed to remove the LAP.

**Fig. 2:**
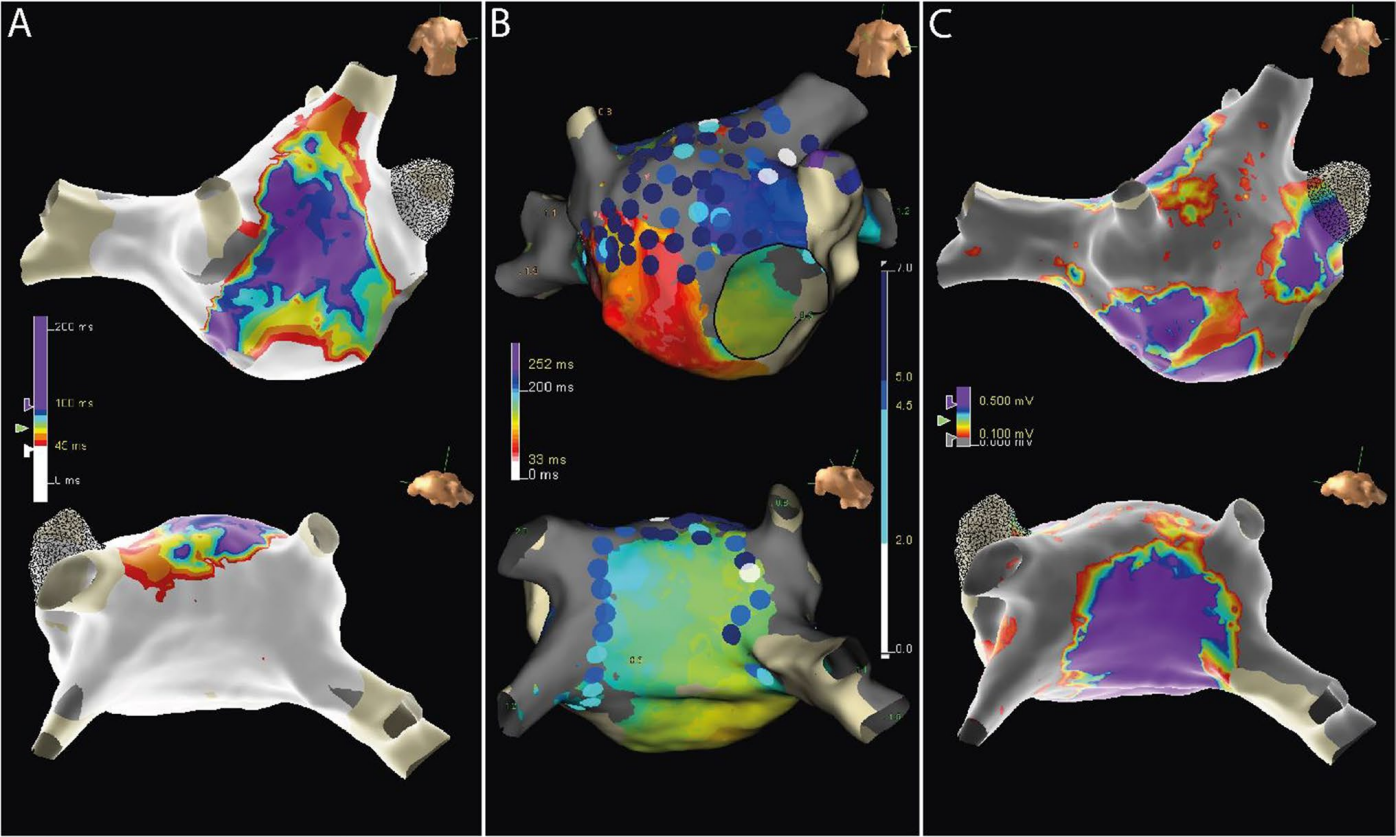
AEDUM map and lesion set. **Panel A** represents the AEDUM of a LA in an example patient with PsAF, RAO and cranial LPO views. White areas exhibit potentials with a duration of <45ms and color-coded areas exhibit potentials with a duration of ≥45ms. **Panel B** shows an activation map of the same LA (LAO and cranial LPO views) after the ablation, demonstrating the block lines across the roof and the anterior wall. White/Cyan/Blue dots show the ablation points, each one color-coded with the LSI™ score. In this example case, the ablation scheme involved antral pulmonary vein isolation, the roof, and part of the anterior wall/inter-atrial septum, extending to the mitral annulus and following the contours of the AEDUM area. **Panel C** shows the remap of the same atrium after the ablation, with a voltage map showing in grey tissue with a voltage <0.1 mV, a color-coded map for voltages between 0.1 and 0.5mV and purple for voltages above 0.5mV. *AEDUM: Atrial Electrogram Duration Map; LA: Left Atrium; PsAF: Persistent Atrial Fibrillation*

Antral circumferential PVI was performed in all Group B patients targeting entrance and exit blocks for each pulmonary vein controlled during SR. In Group B_2_, ablation was extended to AEDUM areas. In the case of “isolated” AEDUM areas, the lesions were extended to anatomical structures or previous lesions to avoid creating a substrate for reentry. For example, in the case of an AEDUM area not involving all the roof regions, the lesion was extended to the two superior veins to avoid roof-dependent atypical atrial flutter. One more example: in the case of AEDUM in the anterior wall near the anterior segment of the mitral valve, the ablation line was prolonged to the mitral annulus to avoid peri-mitral atrial flutter. See Fig. 2*–* Supplemental Figure 1 for a representative example.

A thorough high-density remap was performed after ablation to verify the homogeneous signals abatement of all the targeted areas. If a sharp and local EGM was recorded inside the targeted area, adjunctive RF applications were delivered and a remap was performed. The block lines were also verified through differential pacing.

All procedures were performed using an open irrigated ablation catheter (Tacticath SE^TM^, Abbott Medical). Lesion formation was guided by LSI™ (5.0 anteriorly and 4.5 posteriorly as a target, using 50W-42° as power settings).

### Clinical follow-up

Clinical follow-up consisted of physical examinations, 12-leads electrocardiograms, and 48 h Holter recordings performed at 1, 3, 6, and, later, every 3 months after ablation. In patients with cardiac implantable electronic devices, arrhythmia recurrence was evaluated by device interrogation at the same time. In addition, after the first visit, every month patients were interviewed by phone to collect information on their heart rhythm and symptoms. Patients were instructed to contact our ambulatory in case of symptoms like palpitations and/or dyspnea. Ablation was deemed successful in the absence of symptomatic or asymptomatic atrial tachyarrhythmia lasting more than 30 seconds, documented on surface ECG, 48-h Holter monitoring, or device interrogation.

### Statistical analysis

The normal distribution of all continuous variables was checked by visual methods (Q-Q plot and histogram) and by the significance test (Kolmogorov-Smirnov normality test and Shapiro-Wilk’s test). For continuous variables, descriptive statistics were provided (number of available observations, mean, standard deviation), while the median [interquartile range (IQR)] was used for non-normal data. Categorical data were described as a number (percentage). The proportion of the categorical variables was compared using a Chi-square analysis or Fisher’s exact test, as appropriate. In the case of non-normally distributed variables, the Mann-Whitney U test was used. The Kaplan-Meier method was used to estimate event-free recurrence in the two groups; differences in each group were compared using log-rank tests. All statistical analyses were performed using STATA statistical analysis software (version 16).

## Results

### Patients’ characteristics

Patients in Group A have a mean age of 32±6 years old, and no history of cardiovascular risk factors or significant diseases. Baseline demographic, clinical, and instrumental data of the study study population are summarized in Table 1. The mean age was 63±8 and 62±7 years in Group B_1_ and Group B_2_, respectively. No significant difference was found between the two B Groups. concerning the age, the prevalence of male sex, cardiovascular risk factors, relevant echocardiographic parameters, or clinical arrhythmic history (Table 1).

**Table 1:** Baseline characteristics of study populations. This table presents baseline characteristics of the study groups (B_1_ and B_2_), comparing demographic, echocardiographic and clinical details between them. Data are presented as mean ± standard deviation or n. (%). No significant difference was found between the two groups. *AEDUM: Atrial Electrogram Duration Map; AF: Atrial Fibrillation; BMI: Body Mass Index; COPD: Chronic Obstructive Pulmonary Disease; IVS: Interventricular Septum; LA: Left Atrium; LEVF: Left Ventricle Ejection Fraction; PVI: Pulmonary Vein Isolation*

	PVI (B_1_) (n. 20)	PVI + AEDUM (B_2_) (n. 20)	*P*-value
Age (years)	62.9 ± 8.2	61.5 ± 6.5	0.598
Male sex	15 (75)	14 (70)	0.613
BMI (Kg/m^2^)	27.8 ± 4.6	28.2 ± 4.1	0.839
Cardiac function and chamber dimensions			
LVEF (%)	58.2±13	62.8 ± 4.7	0.250
LA Volume (ml)	79.1 ± 28.9	81.7 ± 28.6	0.938
Indexed LA Volume (ml/m^2^)	38 ± 12	45 ± 18	0.3823
Clinical Data			
Hypertension	11 (55)	15 (75)	0.259
Dyslipidaemia	8 (40)	3 (15)	0.229
Diabetes	1 (5)	3 (15)	0.565
Smoking	4 (20)	1 (5)	0.355
Stroke	0 (0)	1 (5)	1
Ischemic cardiomyopathy	1 (5)	0	1
Peripheral artery disease	1 (5)	0	1
COPD	4 (20)	1 (5)	0.355
Persistent AF duration			
< 6 months	3 (15)	1 (5)	0.356
6-12 months	10 (50)	11 (55)	1
>12 months	7 (35)	8 (40)	1

In Group B_1_ patients, 15% had AF persistence < 6 months; 50% between 6 and 12 months, and 35% ≥12 months. In Group B_2_ patients, 5% had AF persistence < 6 months; 55% between 6 and 12 months, and 40% ≥12 months. No significant difference was found in each AF duration category between Groups B_1_ and B_2_, as reported in Table 1.

Electro-anatomic maps during sinus rhythm

The average number of points used in the 3D LA map was comparable in Groups A, B_1,_ and B_2_ (2979±478; 3110±942 and 2983±381, respectively; *p*-value: 0.642). In 37 out of 40 patients (92%) in Group B, SR was restored after ECV and a map in SR was feasible. In the remaining 3 patients (8%, 1 belonging to Group B_1_ and 2 to Group B_2_), the SR could not be achieved with the first ECV and a second ECV was successfully performed after PVI. The mean heart rate during mapping was 71 ± 8 bpm in Group A; 68 ±6 bpm in Group B_1_ and 62 ± 8 bpm in Group B_2_ (*p*-value: 0.482).

### Bipolar voltage

**Fig. 3:**
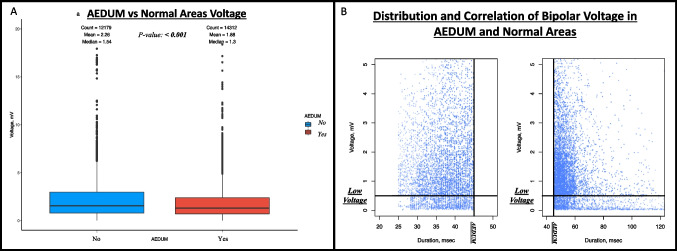
Bipolar voltage and EGM duration in AEDUM and normal atrial areas. **Panel A:** Bipolar voltage in AEDUM and normal atrial areas. **Panel B:** Distribution and correlation of Bipolar Voltage in AEDUM and non-AEDUM areas. *AEDUM: Atrial Electrogram Duration Map*

The findings obtained from SR maps are shown in Table 2. The overall bipolar voltages in the LA were normal in all the patients with similar values in the three groups, [3.69 ± 2.4 mV, 3.07 ± 1.8 mV, and 3.03 ± 1.96 mV in Groups A, B_1_, and B_2_ respectively (*p*-value: 0.891)]. Considering patients in the two B Groups, the overall bipolar voltage in the AEDUM areas was lower than in the remaining left atrial surface [median: 1.30 mV (IQR: 0.71–2.38 mV) vs. 1.54 mV (IQR: 0.79–2.97mV); *p*-value: <0.001) (Fig. 3, Panel A). In particular, in three patients (1 in Group B_1_ and 2 in Group B_2_), a LVA was found in the LA. These LVAs were found in one case on the roof toward the right superior pulmonary vein and two cases in the anterior wall.

**Table 2:** Electroanatomic maps collected during sinus rhythm. This table presents mapping data of the control and study groups. Data are presented as mean ± standard deviation or n. (%). No significant difference was found between the study (B_1_ and B_2_) groups. *AEDUM: Atrial Electrogram Duration Map; EGM: Electrogram*

	Group A (n. 15)	Group B_1_ (n. 20)	Group B_2_ (n. 20)	*P*-value between B_1_ and B_2_
Number of points used	2979±478	3110±942	2983±381	0.642
Mean voltage (mV)	3.69 ± 2.4	3.07 ± 1.8	3.03 ± 1.96	0.891
Patients with areas of low voltage (%)	0	1 (5%)	2 (10%)	1
EGM Duration				
Patients with AEDUM areas (EGM>45ms)	0	20 (100%)	20 (100%)	1
AEDUM Areas extension (cm^2^)	0	22.3 ± 9.1	21.8 ± 8.2	0.447
Overall EGM duration (ms)	34.2 ± 3.7	48.6 ± 15.8	49.5 ± 16.4	0.858

### EGM duration

**Fig. 4:**
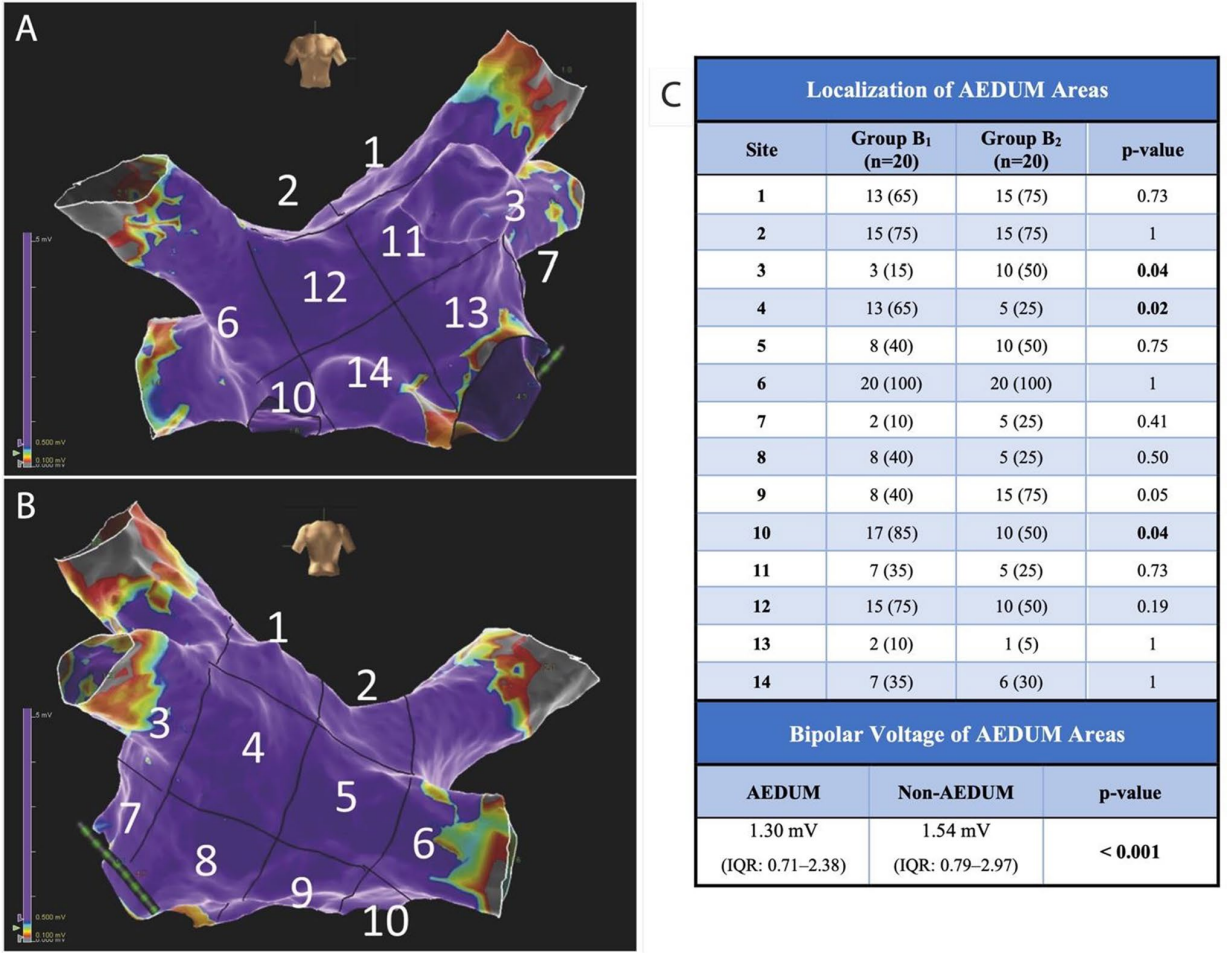
Localization and bipolar voltage of the AEDUM areas. **Panels A and B** show the localization (anterior and posterior view) of the 14 segments we divided the LA into. **Panel C**. shows the difference in the localization of the AEDUM areas in the B_1_ and B_2_ subgroups. For Group B_2_ was reported the distribution of the ablated AEDUM areas. Values are n (%) or mean (Interquartile Range). *AEDUM: Atrial Electrogram Duration Map; IQR: Interquartile Range*

As depicted in Supplemental Figure 2 the mean overall EGMs duration recorded in the LA was higher in Group B than in Group A (49.0 ± 16.2 msec vs 34.2 ± 3.7 msec; *p*-value: <0.001). No significant difference was found in the means of EGM duration between Group B_1_ and B_2_ (48.6 ± 15.8 msec vs 49.5 ± 16.4 msec, respectively; *p*-value: 0.858) (Table 2). All the patients included in Group B presented at least one AEDUM area, while none was found in Group A. In particular, the mean EGM duration was significantly longer in the AEDUM areas than in the remaining left atrial surface (58.2 msec ± 16.9 msec vs 38.3 ± 4.7 msec; *p*-value: <0.001). In *Ps*AF patients, the mean cumulative AEDUM area was 21.8 ± 8.2 cm^2^, with no significant difference between Group B_1_ and Group B_2_ (22.3 ± 9.1 cm^2^ vs 21.2 ± 7.2 cm^2^; *p*-value: 0.45). The overall distribution of the AEDUM area was reported in Fig. 4.

### Correlation between EGM duration and bipolar voltage

**Fig. 5:**
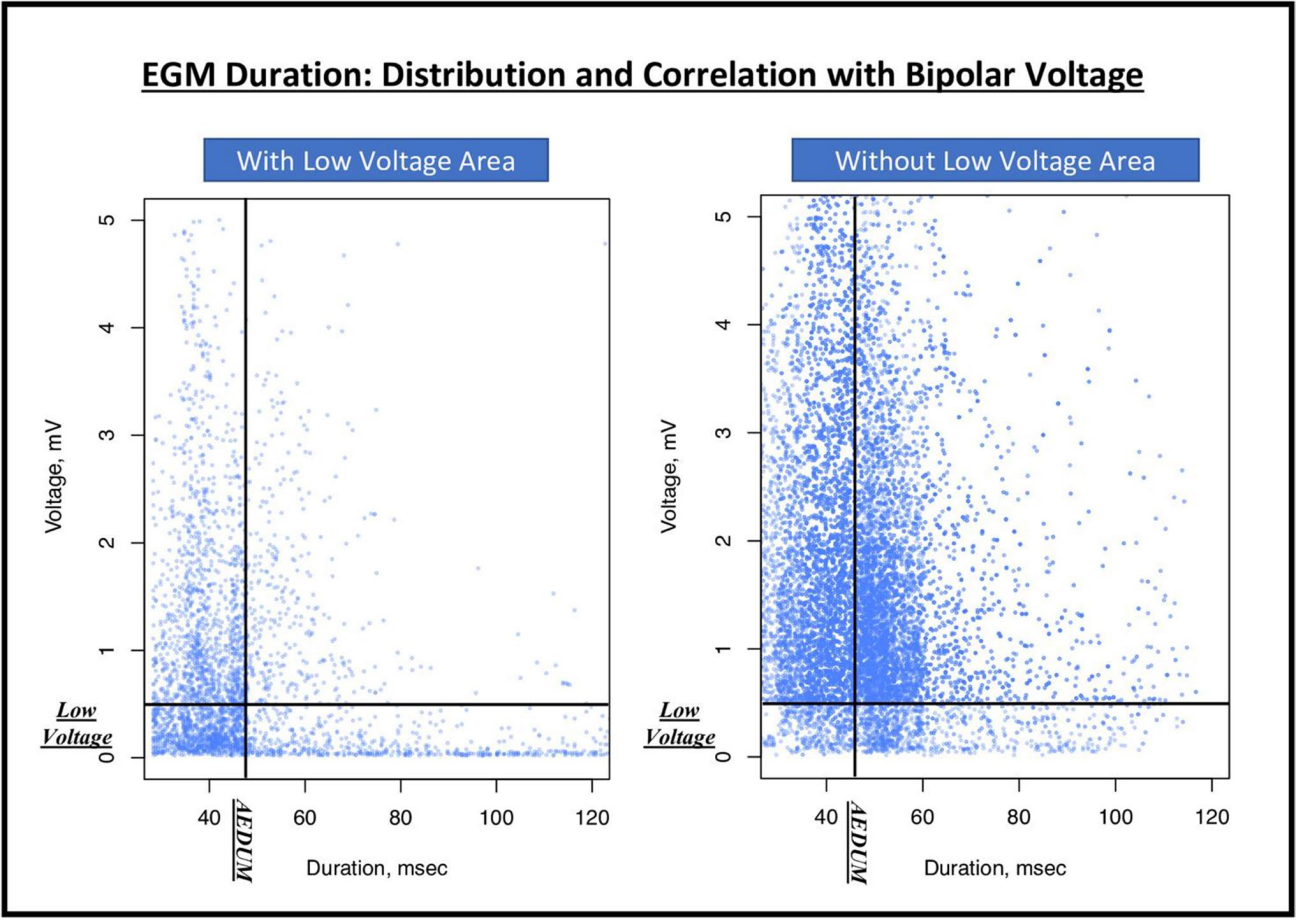
EGM duration: distribution and correlation with bipolar voltage in patients with and without low voltage area. *AEDUM: Atrial Electrogram Duration Map; EGM: Electrogram*

The overall median EGM duration in the 37 patients without LVA was longer than in the 3 patients presenting LVA [median EGM duration: 46.4 msec (IQR: 40.2–53.3 msec) vs 42.9 msec (IQR: 37–52.8 msec); *p*-value: <0.001]. Similarly, the overall median bipolar voltage was higher in patients without LVA [median bipolar voltage: 1.55 mV (IQR: 0.86–2.90 mV) vs 0.47 mV (IQR: 0.16–1.11 mV); *p*-value: <0.001] (Fig. 5). As previously described, the overall bipolar voltage of the AEDUM areas was lower than the remaining surface of the left atrium; in particular, the total acquired points in the AEDUM zones with bipolar voltage < 0.5 mV were 14.2% vs 12.4% (*p*-value: <0.001) in the normal atrial zones (Fig. 3, Panel B).

### Ablation

Ablation of the accessory pathway was successfully performed in all patients in Group A.

Circumferential PVI was obtained in all Group B patients, and a bi-directional block at the PV antrum was verified during SR. In Group B_2_, AEDUM-guided ablation was performed in addition to antral PVI. Periprocedural characteristics were summarized in Supplemental Table 1; no complications were observed either in Group B_1_ or B_2_ related to the procedure either in the acute phase or during follow-up. The roof region was ablated in all patients of Group B_2_ (20 out of 20 patients, 100%) being a site of the AEDUM area. The PW ablation (box isolation) was performed in 7 of 20 patients (35%) while RF was delivered in the anterior septum and the anterior wall in 13 of 20 (65%) patients. In 4 of 20 patients (20%), AEDUM areas were found either in the PW, the roof and anterior wall. In these cases, an extensive ablation was arranged and a comprehensive description of the AEDUM areas ablated was reported in the Fig. 4. Fig. 2 and Supplemental Figure 1 reported the variable and tailored operative schemes.

In one patient (5%) in Group B_2_ was not possible to obtain a complete signal abatement in the area on the anterior aspect of the roof, toward to right superior pulmonary vein.

### Clinical follow-up

**Fig. 6:**
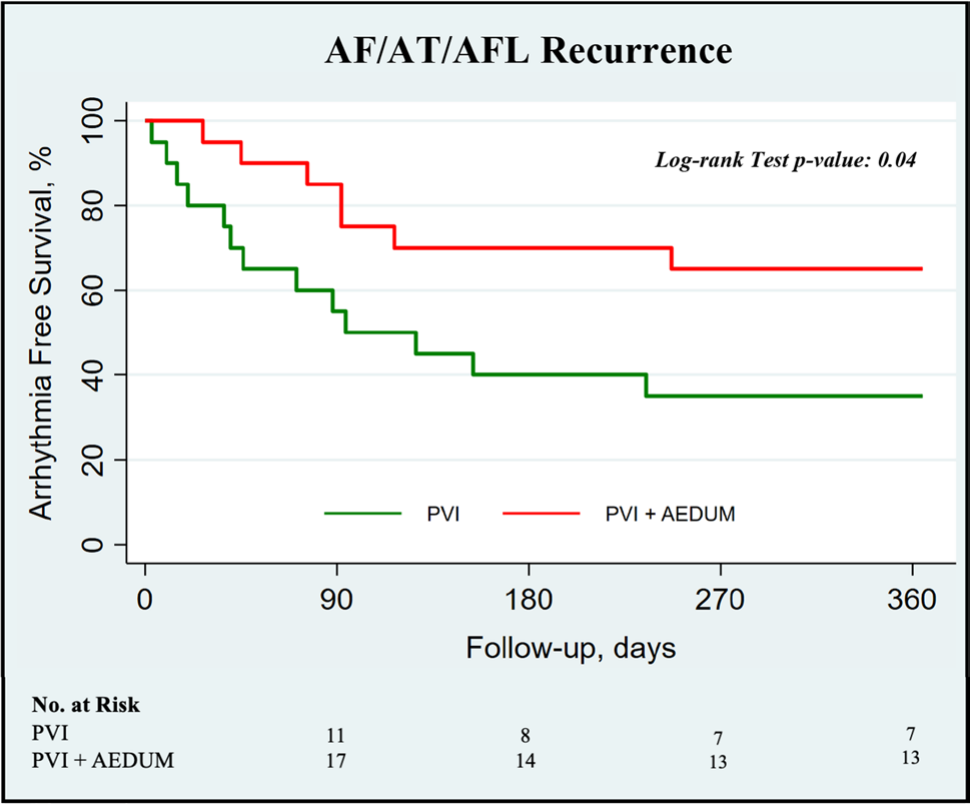
Kaplan Meier Curve comparing disease-free survival between PVI only group (B_1_) and PVI + AEDUM group (B_2_). *AEDUM: Atrial Electrogram Duration Map; AF: Atrial Fibrillation; AFL: Atrial Flutter; AT: Atrial Tachycardia. PVI: Pulmonary Vein Isolation*

The median follow-up of our cohort was 511 days (376–845 days). At 12 months, a documented recurrence of AF or atrial tachycardias lasting longer than 30 s had occurred in 7 of 20 patients (35%) assigned to PVI + AEDUM group and in 13 of 20 patients (65%) assigned to PVI alone group (log-rank *p*-value=0.04) (Fig. 6). In 9 (22.5%) of 40 patients with cardiac implantable electronic devices, arrhythmia recurrence was evaluated by device interrogation; the AF/AT episodes for each patient were reported in Supplemental Figure 3.

### Histology analysis from atrial biopsy

**Fig. 7:**
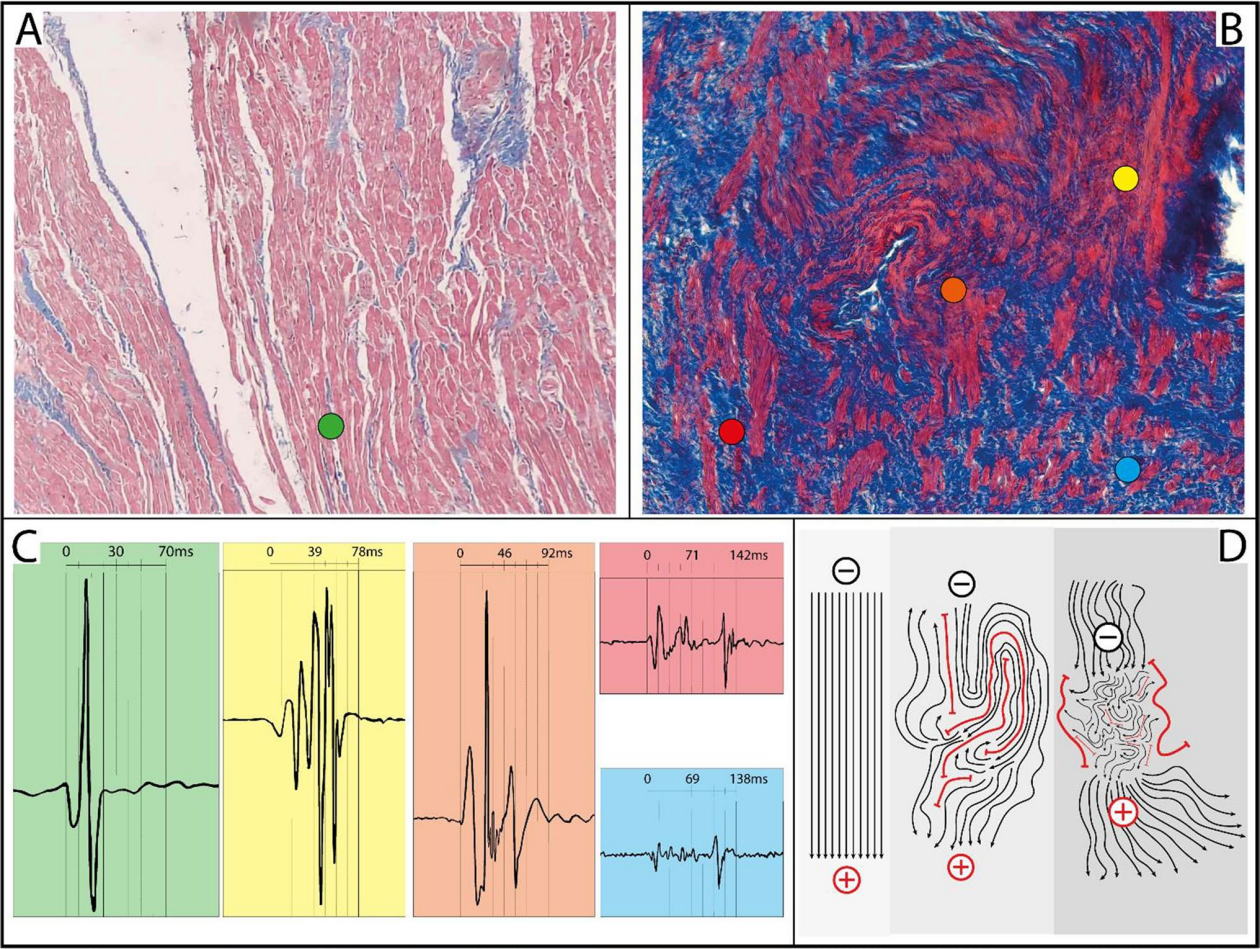
Histological findings in the AEDUM areas. **A and B panels:** tissue sample from atrial biopsies taken in the RA (panel A) and LA (Panel B) of the same patient with PsAF, prepared applying the Masson trichromic staining protocol. Red color shows the presence of cardiomyocytes, while blue color highlights the presence of collagen. Note the high prevalence of collagen fibers and muscular bundles disarray in the LA versus the RA. **Panel C:** Sample digitalized potentials obtained from the atria of the same patient, with their relative color-coded dots showing a hypothetical electro-histological correlation with panels A and B. **Panel D:** Schematic representation of depolarization vectors (arrows) is shown to exemplify the possible dynamics of the propagation which could be the basis for the recorded signal morphologies: the first example (left) shows conduction over healthy tissue, the second (middle) one shows conduction over bundles of healthy fibers with non-uniform anisotropism and modest presence of conduction block phenomena (red lines); the third one (right) shows conduction over a un-healthy tissue with small and non-uniformly anisotropic fibers, with possible high presence of collagen and functional block phenomena (red lines). *AEDUM: Atrial Electrogram Duration Map; LA: Left Atrium; PsAF: Persistent Atrial Fibrillation; RA: Right Atrium*

One patient of Group B_1_ during the post-ablation follow-up period had a worsening mitral regurgitation that required mitral valve replacement. During the surgical intervention, punch biopsies of atrial tissue were obtained guided by the previous endocardial map; a continuous running 5-0 Prolene suture closure was performed to avoid bleeding. One specimen was taken in the RA, at the surgical access site (Fig. 7–- Panel A), and another one in the region of the LA roof area (Fig. 7 – Panel B), corresponding to an AEDUM area previously observed during PVI procedure. The histologic evaluation was performed by applying the Masson trichromic staining protocol. Abundant collagen deposition within bundles between myocytes as well as muscle fibers’ disarray were found in the AEDUM area of the LA, while physiological histology was found in the RA.

## Discussion

This study is the first clinical report analyzing functional LA substrate through a new map displaying the spatial distribution of bipolar EGMs’ local durations. This strategy evidences the areas characterized by local conductive abnormalities, that are likely to be relevant for AF maintenance and thus may be targeted for tailored treatment of *Ps*AF.

The main findings of the present study are:

- The LA of patients with *Ps*AF is characterized by discrete areas of inhomogeneous conduction during SR revealed by the AEDUM map.
- The AEDUM areas are mainly located in zones with voltage > 0.5 mV.
- An anatomical ablation approach targeting the AEDUM areas beyond PVI resulted in improved AF-free survival when compared to a PVI alone approach.

### Distribution of AEDUM areas and ablation schemes

The regions of slow and inhomogeneous conduction mostly involved in our study population are the LA roof (91% of the cases) and the anterior wall (82%). The common involvement of the LA roof is in accordance with our previous findings demonstrating that the LA roof is the first region of slow conduction in patients with paroxysmal AF [18]. A conductive dysfunction involving the roof and anterior wall of LA, could involve also Bachmann’s bundle and may explain an increased P wave duration and interatrial conduction time in patients with PsAF [20]. Surprisingly, the PW was involved in one-third of the cases (36%) and this result could justify the discordant clinical outcome after PW isolation [21].

Further, the left isthmus region was found to be the site of an AEDUM area only in a minority of the cases (9%) and this is the reason why we did not perform substrate modification at this level in our population. Anyway, to avoid the risk of a peri-mitral flutter after extensive ablation beyond PVI, in the patients presenting an AEDUM area in the anterior wall (13 out of 20, 65%) we extended the anterior lesions from the PVI line to the mitral annulus.

Of importance, the ablations we performed were not just linear lesions but bands covering the whole abnormal functional substrate in order to eliminate the sites of abnormal activation. Giving these characteristics, our procedural and RF time were usually prolonged than other anatomical approaches (PW isolation, Marshall Alcholization). On the other hand, the increased time spent for the detection and the ablation of the AEDUM areas may represents a tailored approach for the elimination of the atrial triggers for the maintenance of *Ps*AF.

### Substrate and anatomically based approaches

Multiple methods, such as complex fractionated atrial electrograms [22] and areas of spatiotemporal EGM dispersion [11], have been developed to target arrhythmia drivers identified with EGM analysis during AF, even if a consistent improvement in arrhythmia free-survival was not reached adopting this approach.

Voltage mapping during AF has also been proposed to identify LVA as evidence of regional tissue disease [23]. At the same time, this analysis might be hampered due to some confounding factors such as functional phenomena like wave-front collisions, responsible for lowering signals’ amplitude.

Voltage mapping analysis during SR instead allows for avoiding the tough interpretation of complex signals recorded during AF [24,25] even if, as shown in the present study, patients with PsAF do not commonly exhibit LVAs [26].

Otherwise, the anatomical approach (roof line and mitral isthmus line) has proven to be superior to PVI-only [27], especially after Marshall’s vein alcoholization [7]. This interventional scheme may avoid the ablation of functional substrate areas maintaining AF and requires a longer procedure duration and X-rays/contrast exposure.

Our strategy could be considered a hybrid approach because anatomical lesions are tailored to each patient’s functional substrate analysis.

### Atrial substrate identification using the AEDUM approach

In bipolar recordings, the time shift between poles is directly proportional to the distance and inversely proportional to the conduction velocity [28]. The EGM duration is indicative of local tissue conduction time especially when measured by dipoles with a small inter-electrode distance that significantly reduces far-field signal components [26,29]. The simultaneous recording of EGMs from multiple dipoles allows for reducing the underestimation of both voltage and EGM duration by following better the main vectors of the wave-front propagation.

The identification of areas with inhomogeneous EGM duration could suggest the presence of augmented non-uniform anisotropism due to molecular and histological remodeling as described in the animal model [30,31].

Atrial tissue remodeling is characterized by patchy and/or interstitial fibrosis, modification of the spatial distribution of intercellular connexins, gap junction remodeling [32] , and muscular tissue disarray [33]. Cellular electrical coupling defects (endomysial fibrosis) are likely to be much more implicated than patchy fibrosis in the atrial disease in the cohort here analyzed [34]. A previous *in vitro* study demonstrated that a significant reduction in electrical coupling may produce arrhythmogenic effects similar to those of severe fibrosis, being able to modify the dynamics of wave propagation, from planar to reentrant [35].

These pathological modifications affect how the depolarization wave spatially propagates between cardiomyocytes, eventually creating a substrate for arrhythmia induction and maintenance. Following these considerations is expected that subjects with longer EGM duration present more inhomogeneous conduction favoring reentry, especially in the event of single or multiple atrial premature beats.

Further, as reported in Fig. 1 the signal recorded presented complex morphology. Rapid and fragmented signal components can be explained by anisotropic conduction, (“zig-zag” conduction) among small muscle bundles while slow components could be due to conduction along large muscle bundles, *each divided* by fibrous strands, with frequent and abrupt changes to their orientation, as in Fig. 7 [28]. Split signals like double potentials were also recorded, representing a block line due to electrical influences during propagation on either side ^36^.

Fractionated signals, which may represent critical sites for atrial arrhythmias can be found also inside a tissue presenting conventional normal voltage ^37^. The present paper supports this concept because the voltage maps are normal in SR in the majority of patients suffering from *Ps*AF. In fact, in the large majority of patients here reported, i.e., 37 out of 40 (93%), LVAs were not found in the LA. Moreover, the bipolar voltage of the AEDUM areas was significantly lower than the remaining left atrial surface. These findings suggest that AEDUM map could identify the early tissue remodeling of the atrial substrate before reaching the cut-off of low voltage area.

## Limitations

This clinical study has some limitations that should be taken into consideration. Firstly, the small size of the study population because this paper should be considered a pilot study. Larger prospective studies are warranted to confirm our results thus increasing evidence on the tailored approach based on the ablation of AEDUM areas. Secondly, EGM duration is a functional phenomenon and the extension of the AEDUM area could be influenced by heart rate, site of stimulation, or atrial extra stimuli. In this study, we did not perform pacing maneuvers to not further prolong the intervention. This could be the objective of future investigations. Additionally, we did not perform atrial magnetic resonance for the detection of scar/fibrosis distribution in the atrium for matching with AEDUM areas. Lastly, we didn’t verify the effects of antiarrhythmic or adrenergic/anticholinergic drugs such as isoprenaline/atropine on the AEDUM areas.

## Conclusions

The measurement of EGM duration during SR, in the atrium of patients with *Ps*AF, identifies discrete areas of inhomogeneous and slow conduction (AEDUM areas). The ablation approach targeting the AEDUM areas results in a more effective strategy to treat *Ps*AF compared with PVI only.

## Supplementary information

ESM 1 (DOCX 7096 kb)

## Abbreviations

AEDUM: atrial electrogram duration map.
AF: atrial fibrillation.
ECV: electrical cardioversion.
EGM: electrogram.
LA: left atrial.
LAP: left-sided accessory pathway.
LVA: low voltage area.
PsAF: persistent atrial fibrillation.
PVI: pulmonary vein isolation.
PW: posterior wall.
SR: sinus rhythm.
