# Supplementary material for: Ablation of persistent atrial fibrillation based on atrial electrogram duration map: methodology and clinical outcomes from the AEDUM pilot study
Source: J Interv Card Electrophysiol. 2024 Jan 11;67(6):1365–76. doi: 10.1007/s10840-023-01721-7 (PMC11379763; doi:10.1007/s10840-023-01721-7)
Supplement: Supplementary file 1 — (DOCX 7096 kb) [file 10840_2023_1721_MOESM1_ESM.docx]

**SUPPLEMENTAL MATERIALS**

**Ablation of Persistent Atrial Fibrillation based on Atrial Electrogram Duration Map: Methodology and Clinical Outcomes from the AEDUM Pilot Study.**

**Expanded Methods**

Atrial Electrogram Duration Map 2

**Supplemental Table**

Supplemental Table 1 3

**Supplemental Figures**

Supplemental Figure 1 4

Supplemental Figure 2 5

Supplemental Figure 3 6

**Expanded Methods**
*Atrial Electrogram Duration Map*

The AEDUM is obtained in review mode after a complete left atrial electroanatomical map. The following steps are made:

1) setup programming for a single bipole of HD Grid:

- Reference: bipole of the HD Grid, using as detection the first signal deflection
- Roving: bipole of the HD Grid (same as the reference), using as a detection the last signal deflection
- Timing reference: coinciding with the first deflection
- Map type: LAT, colors distributed with auto-color tool

2) Setting of the automap tool aimed to analyze only the atrial signals

3) Activation of the Turbomap tool for each HD Grid dipole

To obtain a high-density map, repeat for 5 or 6 different bipoles using the Turbomap tool. The choice of the HD Grid bipoles is not mandatory but it is recommended to choose bipoles of different splines (across and along).

When the AEDUM area is automatically delimited by the system, a careful manual check of the points projected on the map inside and around the AEDUM area is made by the Engineer. It is necessary to be accurate to control especially the final component of the analyzed EGM which often has a very low amplitude.

The mean time needed by an expert Engineer is about 30 minutes to create a controlled AEDUM map.

**Supplemental Table**

**Supplemental Table 1. Periprocedural Data.** Values are n (%) or mean ± standard deviation. *AEDUM: Atrial Electrogram Duration Map; PVI: Pulmonary Vein Isolation.*

|  | **PVI (B_1_)**  **(n. 20)** | **PVI + AEDUM (B_2_)**  **(n. 20)** | **P-Value** |
| --- | --- | --- | --- |
| General anaesthesia | 20 (100) | 20 (100) | 1 |
| Procedural duration, min | 116.7 ± 34.3 | 163.6 ± 43.2 | <0.001 |
| Fluoroscopy time, min | 11.2 ± 3.4 | 12.7 ± 4.1 | 0.08 |
| Radiofrequency time, min | 21.2 ± 12.3 | 36.8 ± 19.4 | <0.001 |
| Major procedure-related events | 0 | 0 | - |
| Minor procedure-related events |  |  |  |
| Groin hematoma | 0 | 1 (5) | - |

**Supplemental Figures**

**Supplemental Figure 1. Schematic representation of the areas selected for treatment.** A and B panel show an antero-posterior projection and a cranial left posterior oblique view of the same left atrium (LA). Green shade highlights the LA roof, magenta shade the posterior wall (PW), yellow the anterior wall (AW) and cyan the antralized portion of pulmonary veins (PV) extending to part of the inter-atrial septum (IAS). Usual ablation scheme involved always the antralization of PVI adding, basing on the AEDUM, lesions in the roof+AW, roof+PW or roof+PW+AW.


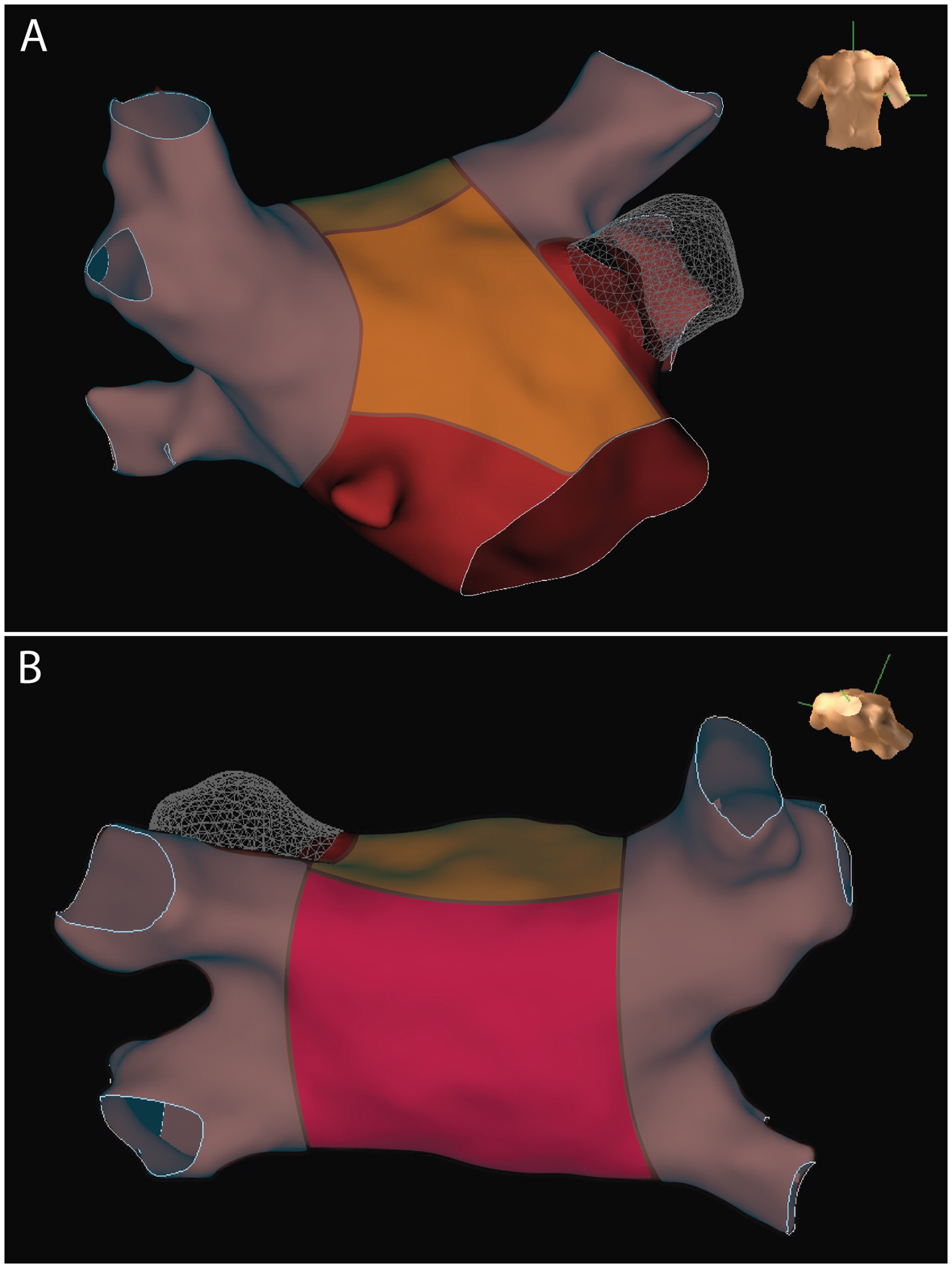


**Supplemental Figure 2. EGMs duration in Group A and in Group B.** *EGM: Electrogram.*

**
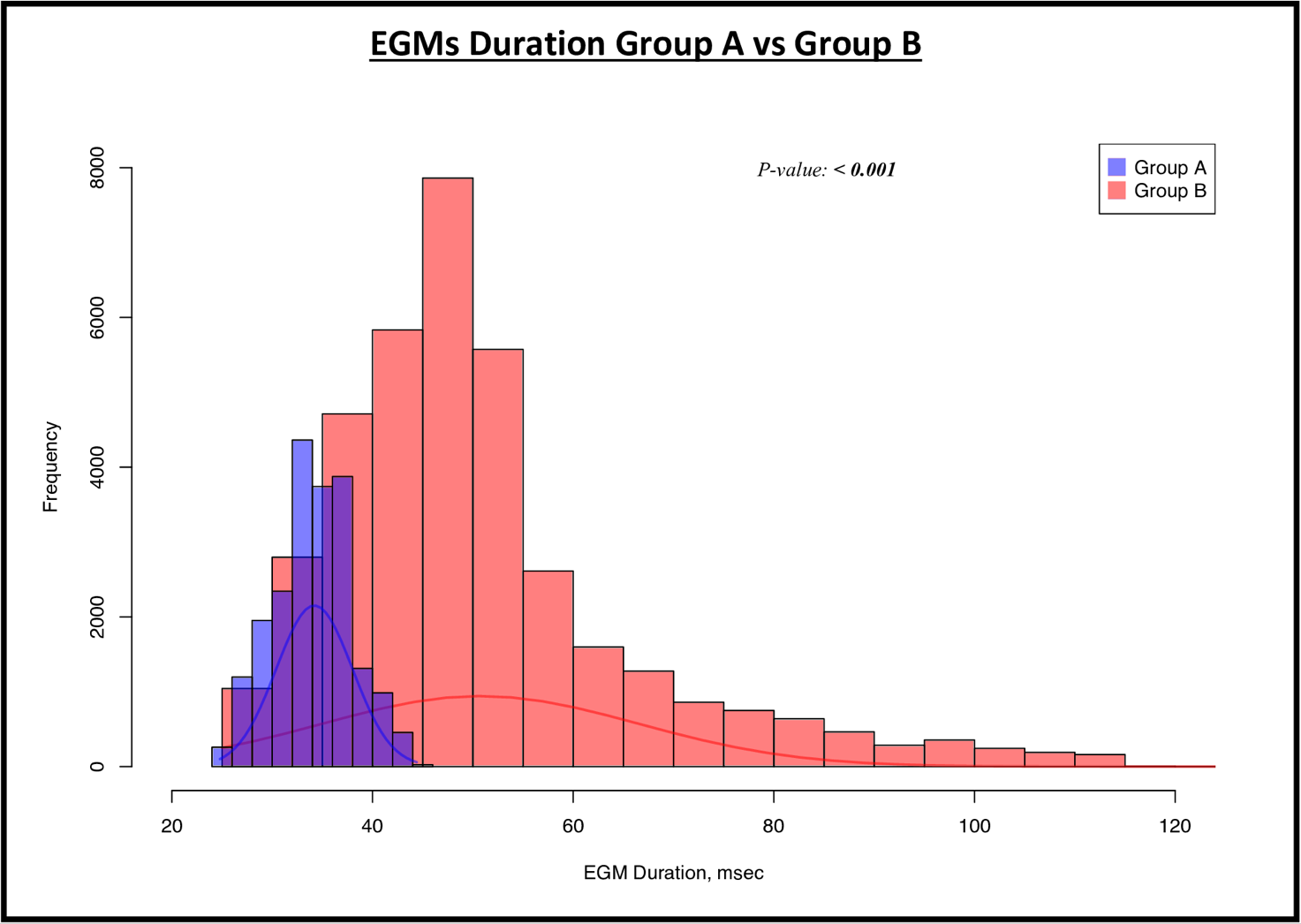
**

**Supplemental Figure 3. Follow-up Timeline for each patient.** *AEDUM: Atrial Electrogram Duration Map; AF: Atrial Fibrillation: AFL: Atrial Flutter; AT: Atrial Tachycardia; CIED: Cardiac Implantable Electronic Device; PVI: Pulmonary Vein Isolation.*
